# N^w^‐Hydroxy L‐Arginine (NOHA): A Promising Biomarker of Response to Neoadjuvant Therapy in the Management of Triple Negative Breast Cancer

**DOI:** 10.1002/cnr2.70609

**Published:** 2026-07-08

**Authors:** Srinidi Mohan, Douglas W. Van Pelt, Jamie G. Saunders, John DiPalazzo, Shelby E. Monahan, Rachel J. Buchsbaum, Cara L. Frankenfeld, Brittany Y. Bertaux, Susan Miesfeldt

**Affiliations:** ^1^ University of New England Portland Maine USA; ^2^ MaineHealth Cancer Care Network Scarborough Maine USA; ^3^ MaineHealth Institute for Research Scarborough Maine USA; ^4^ Tufts University School of Medicine and Tufts Medical Center Boston Massachusetts USA

## Abstract

**Background:**

Oncologists lack effective point‐of‐care biomarkers to rapidly adapt curative‐intent triple negative breast cancer (TNBC) neoadjuvant therapy (NT). Previous work reveals that N^w^‐hydroxy‐L‐Arginine (NOHA) is an easily measured and sensitive plasma‐based biomarker for breast cancer hormone receptor status, disease stage, and grade, with reduced levels correlating with the presence of estrogen receptor negative and high‐risk disease.

**Aims:**

This pilot study examined NOHA's potential as a marker of both TNBC disease presence and treatment exposure to curative intent TNBC NT, including evaluation of the relationship between longitudinal NOHA normalization and measures of pathologic response.

**Methods and Results:**

NOHA concentration was assessed pre‐NT and then longitudinally at five timepoints, including pre‐ and post‐operatively, among 31 TNBC patients undergoing definitive surgery following receipt of NT. NOHA concentrations were low pre‐NT and increased progressively toward healthy control levels during and after neoadjuvant therapy and post‐operatively in all patients, yet showed no difference comparing those achieving pathologic complete response versus pathologic partial response or stable disease. Levels did not vary based on receipt of pembrolizumab, clinical characteristics, or presence of non‐cancer comorbidities.

**Conclusion:**

These findings support NOHA's potential as a marker of disease presence and treatment exposure rather than differential response. Larger validation studies are needed to determine whether serial levels can be used to optimize treatment decision‐making or improve outcomes in high‐risk populations.

## 1 Introduction

Disease‐free and overall survival in triple negative breast cancer (TNBC) are linked to early disease detection and pathologic complete response (pCR) to neoadjuvant therapy (NT) [1, 2]. The addition of PARP and immune checkpoint inhibitors has pushed NT pCR rates to ~60% [3, 4, 5]. Despite these treatment advancements, there is a gap in reliable blood‐based biomarkers to rapidly adapt and optimize NT decision‐making throughout the course of therapy, limiting the achievement of pCR and risking exposure to ineffective agents that cause adverse events [1, 3].

Emerging evidence suggests that circulating N^w^‐hydroxy‐L‐Arginine (NOHA) reflects the inflammatory and metabolic reprogramming that occurs with breast cancer progression [6]. NOHA is a stable intermediate formed during nitric oxide synthase‐2 (NOS2)‐mediated conversion of L‐arginine to nitric oxide [7]. Aggressive high‐grade breast cancers however, including TNBC subtypes, exhibit elevated arginine consumption to support tumor proliferation, limiting available arginine substrate for NOS2‐mediated NOHA formation and efflux into systemic circulation [8]. Consequently, low plasma NOHA levels may serve as a systemic indicator of tumor‐driven arginine depletion and disease burden [9].

In support of this conceptual framework, we have demonstrated that plasma NOHA concentration distinguishes ER negative (ER‐) from ER positive (ER+) disease among breast cancer‐affected individuals [8, 10]. Specifically, NOHA levels of < 4 nM, 4–8 nM and > 8 nM reflect ER−, ER+ and healthy control levels, respectively. Furthermore, NOHA levels correlate with disease stage, grade, and molecular phenotype [8].

NOHA is easily measured in plasma with our patented [11, 12] and analytically validated enzyme‐linked immunosorbent assay (ELISA) [13], demonstrating sensitivity equivalent to liquid chromatography‐mass spectrometry (LC–MS) in patient plasma samples. The simple, yet sensitive ELISA enables NOHA measurement without the need for expensive and technically complex LC–MS methodology, further supporting its potential clinical utility as a minimally invasive and accessible breast cancer biomarker.

Building on these cross‐sectional observations, the present pilot study tests whether serial NOHA monitoring can serve as a dynamic indicator of NT response in TNBC. We hypothesized that reduced plasma levels would reflect the presence of high‐risk disease, and that longitudinal NOHA normalization would track both treatment exposure and clinical and pathologic response to curative intent NT in TNBC [8, 14]. To test this hypothesis, this exploratory work examined pre‐treatment and longitudinal NOHA levels among individuals undergoing TNBC NT, and compared differences based on measures of pathologic response.

## 2 Patients and Methods

### 2.1 Study Setting

Study participants were recruited through the MaineHealth Cancer Care Network (MHCCN), including Maine Medical Center (MMC), and three additional network‐affiliated hospitals within Maine, as well as Tufts Medical Center (Boston, MA) from December 2018 through March 2024. All NOHA testing was performed in Dr. Mohan's laboratory at the University of New England, with laboratory personnel blinded to all clinical data until data analysis. The MMC Institutional Review Board (IRB) approved the study protocol and procedures. All participants provided written informed consent before enrollment. Methodologies conformed to Declaration of Helsinki standards.

### 2.2 Study Participants

Eligibility criteria included patients ages 18 years or older, with newly diagnosed TNBC defined by immunohistochemistry (IHC) as ER negative (< 1% expression) or low positive (1%–9% expression), PR negative (< 1% expression), or low positive (1%–9% expression), HER2 negative by IHC or fluorescence in situ hybridization (FISH), with measurable disease, and scheduled to undergo curative‐intent NT. Although current American Society of Clinical Oncology/College of Anatomic Pathology (ASCO/CAP) guidelines define hormone receptor negativity as < 1% ER and PR expression, patients with tumors exhibiting low hormone receptor expression (1%–9%) were included since accumulating evidence suggests that tumors with low ER or PR expression frequently demonstrate characteristics and clinical behavior similar to TNBC, including reduced endocrine responsiveness and comparable pCR rates to NT [15, 16, 17, 18, 19, 20]. Inclusion of this subgroup reflects real‐world treatment paradigms and was intended to maximize exploratory evaluation of NOHA as a biomarker in those with aggressive disease. Individuals with multiple primary breast cancers were eligible if at least one lesion met TNBC criteria. We report only on subjects taken to definitive surgery following NT.

### 2.3 Sample and Data Collection

Whole blood was collected via venipuncture from subjects at five time points throughout the course of NT: (1) within 7 days prior to NT initiation (Pre‐NT), (2) 4–8 weeks after start of NT (NT 1), (3) 10–14 weeks after the start of NT (NT2), (4) 1–3 weeks after completion of NT, and (5) 2–7 weeks after surgery. Sample collection was allowed to occur outside of these windows in the event of clinic visit delays. Blood samples were collected in EDTA tubes and plasma supernatant was isolated via ultracentrifugation for 10 min at 1000–2000×*g*, within 15 min of blood collection. Plasma samples were stored at −65°C until analysis.

Basic sociodemographic and clinical data were collected at each site by clinical trial coordinators via patient self‐report and electronic record review. Data was de‐identified, coded, and entered into a secure REDCap database.

### 2.4 Plasma NOHA Analysis

Plasma NOHA concentration was measured for each sample in duplicate using our established ELISA protocol, as previously described [13]. Briefly, BSA–NOHA–coated strips were washed three times with 200 μL of 1X PBS. Each well received 100 μL of a complex mixture composed of 80 μL of either sample lysate or standard, combined with 20 μL of NOHA–mAb at 8 ng/mL subsequently incubated for 1 h at 25°C. Contents were then discarded and wells washed eight times with 200 μL of PBS. An HRP‐conjugated secondary antibody (Abcam, Cambridge, MA), diluted 1:20000 in PBS, was added (100 μL/well) and incubated for 1 h at 25°C. Following another set of eight washes, 100 μL of TMB substrate was added and incubated in the dark for 10 min. The reaction was terminated with 100 μL of 0.1 N HCl. Absorbance was measured at 450 nm using a VersaMax spectrophotometer (Molecular Devices, NH). A second‐order polynomial standard curve was generated, at *R* ^2^ ≥ 0.99, for standard curve fitting and NOHA quantification.

### 2.5 Statistical Analysis

A linear mixed model was used to assess the effect of treatment (NT and surgery) and treatment response, that is, complete pathologic response to therapy (pCR+) versus those showing partial pathologic response or stable disease (pCR−), on NOHA plasma levels. The model's fixed effects are time, treatment response, and their interaction; its random effects are the participants. The model was fitted using the lme4 package in *R*, applying restricted maximum likelihood estimation and an unstructured covariance matrix. Model assumptions were verified by inspection of residuals and *Q*–*Q* plots, and missing data were handled under the missing‐at‐random assumption via maximum likelihood.

Univariable regression models were used to assess association between pre‐treatment NOHA levels and age, race, non‐cancer comorbidities, cancer grade, and stage. Also, univariable regression models were calculated to assess the association between receipt of pembrolizumab and NOHA levels post‐NT and after surgery. *p* < 0.05 was considered statistically significant. All data processing and statistical analyses were performed with the R statistical computing software (version 4.2.1; R Core Team 2022).

## 3 Results

### 3.1 Subject Characteristics and Pathological Response

**FIGURE 1:**
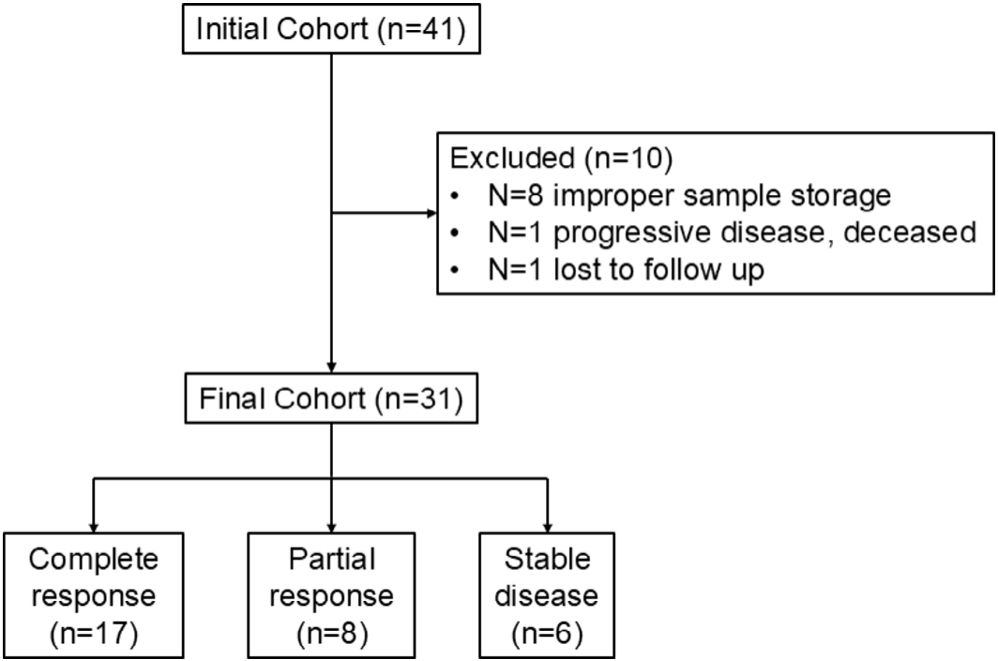
Consort diagram.

Ten of forty‐one enrolled patients were excluded: eight due to improper plasma sample storage, one due to incomplete data caused by interval progressive disease and death, and one lost to follow up (Figure 1). The remaining 31 participants were all female, mostly white (94%, *n* = 29), with about half exhibiting one or more non‐cancer co‐morbidities (48%, *n* = 15), most often hypertension (Table 1). Most had clinical stage II and III disease, with 35% (*N* = 11) exhibiting stage III disease. The majority had grade 3 disease (74%, *n* = 23). One had two primary tumors, including a triple‐negative and an ER negative/PR positive lesion. All TNBC lesions were ER negative, two were PR low‐positive. Seventeen (55%) exhibited a complete NT pathologic response (pCR+) while 14 exhibited partial response (*n* = 8) or stable disease (*n* = 6), (pCR‐), as assessed by chart review of clinical notes and pathology reports (Table 1).

**TABLE 1:** Subject characteristics.

	*n* = 31, *n* (%)
Age (years)	57 (41.69)
Gender	
Female	31 (100%)
Race and Ethnicity	
Black or African American	1 (3%)
Unknown	1 (3%)
White	29 (94%)
Ethnicity	
Non‐Hispanic	28 (90%)
Unknown	3 (10%)
Comorbidity count	
0	16 (52%)
1	7 (23%)
> 1	8 (26%)
Stage	
I	3 (10%)
II	17 (55%)
III	11 (35%)
Grade	
2	4 (13%)
3	23 (74%)
None	4 (13%)
Surgical response	
Complete response (pCR+)	17 (55%)
Partial response (pCR‐)	8 (26%)
Stable disease (pCR‐)	6 (19%)

### 3.2 Neoadjuvant Therapy Received

This study originated in 2018 before the incorporation of pembrolizumab in the neoadjuvant treatment of TNBC, so most subjects initially enrolled received dose‐dense anthracycline‐taxane based NT (*n* = 9); or an alternative taxane‐based regimen (*n* = 2); 3 received adjuvant capecitabine. A total of 20 (65%) participants received combined chemotherapy and pembrolizumab.

### 3.3 NOHA Plasma Concentration and Neoadjuvant Treatment Response

**FIGURE 2:**
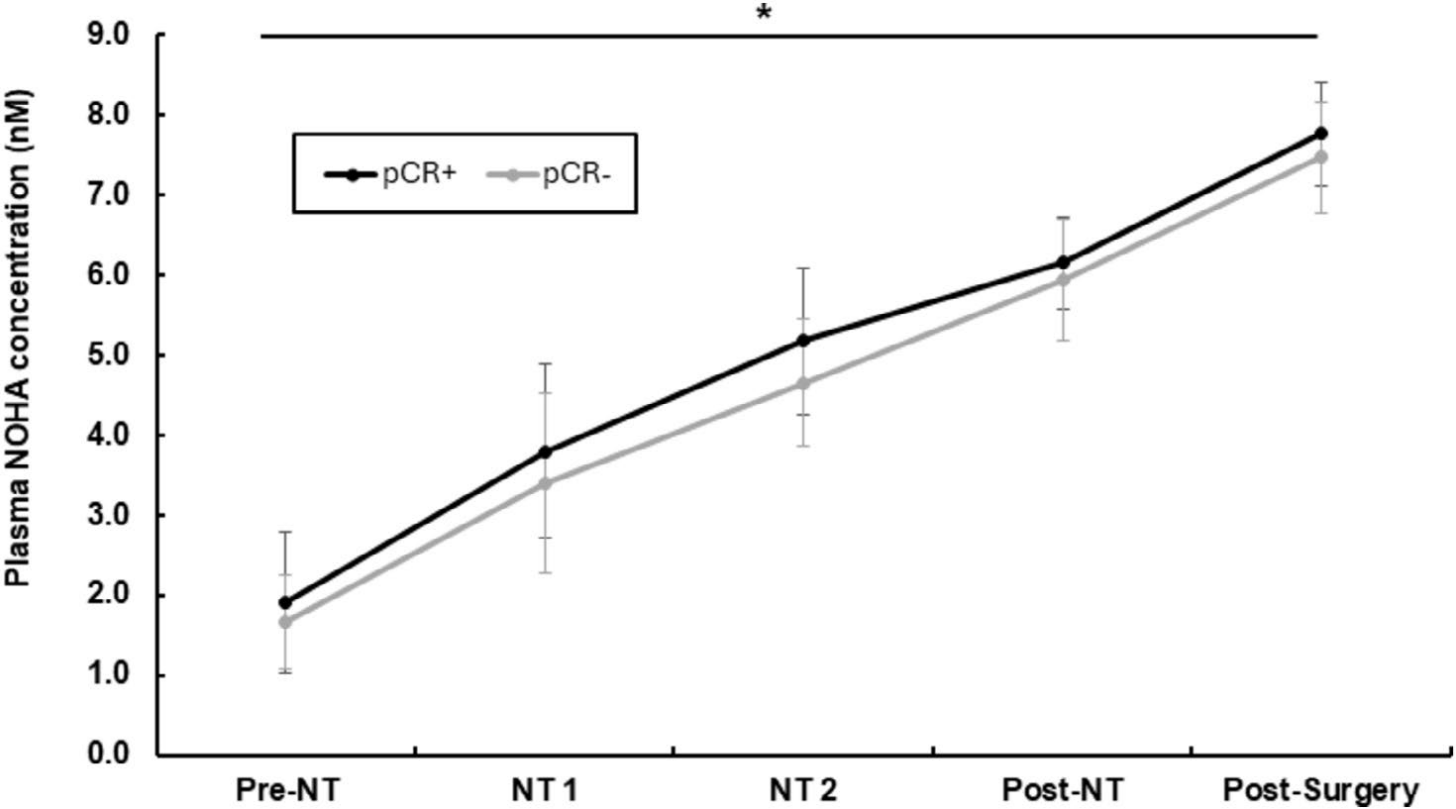
Longitudinal plasma NOHA concentration by surgical response. pCR+ (*n* = 17) included those exhibiting a complete pathological response to NT. pCR‐ (*n* = 14 total) were patients that exhibited partial pathological response (*n* = 8) or stable disease (*n* = 6). Pre‐NT = within 7 days prior to NT initiation, sample *n* = 29; NT 1 = 4–8 weeks after start of NT, sample *n* = 28; NT 2 = 10–14 weeks after the start of NT, sample *n* = 27; Post‐NT = 1–3 weeks after completion of NT, sample *n* = 27; Post‐surgery = 2–7 weeks after surgery, sample *n* = 25. Data analyzed using a linear mixed model. *p < 0.001, main effect of treatment with no differences between groups (*p* = 0.374) or treatment: Group interaction (*p* = 0.935).

Pretreatment NOHA levels were low (Mean ± SD = 1.80 ± 0.76 nM) and significantly increased throughout NT (Post‐NT Mean ± SD = 6.06 ± 0.66 nM) and following surgery (Post‐Surgery Mean ± SD = 7.65 ± 0.67), towards healthy levels (8 nM), in all subjects (Figure 2; *F*(4, 98.15) = 162.69, *p* < 0.001, main effect for treatment; Figure S1). There were no differences in NOHA across all timepoints comparing pCR+ with pCR‐ (Figure 2; *F*(1, 96.85) = 0.79, *p* = 0.374, no effect of group) and no treatment: group interaction (Figure 2; *F*(4, 98.23) = 0.21, *p* = 0.935).

### 3.4 NOHA Plasma Concentration and Other Clinical Factors

There was no correlation between pretreatment or post‐surgery NOHA levels and clinical characteristics, administration of pembrolizumab, or the presence of non‐cancer comorbidities (Table S1).

## 4 Discussion

This exploratory pilot study of individuals with TNBC undergoing curative intent NT demonstrated that plasma NOHA concentrations correlate with the presence of disease and rise longitudinally toward healthy‐control levels during and after treatment, yet showed no correlation with achievement of pCR in this small cohort. Specifically, consistent with our earlier work, all study participants had pretreatment NOHA levels well below 4 nM (Mean 1.80 nM), corresponding with the presence of ER negative, high‐risk disease. NOHA normalization (NOHA approaching 8 nM) was demonstrated in all subjects throughout the course of NT, reaching an average of 6.1 nM following completion of NT and 7.7 nM postoperatively. Our inability to demonstrate a difference in NOHA normalization in those attaining a pCR versus < pCR is suspected to reflect the small sample size, coupled with treatment delay‐imposed variability in the collection and timing of interval blood draws. It is noteworthy that there was no relationship between pre‐treatment NOHA levels and non‐cancer comorbidities, suggesting that reduced NOHA levels are reflective of the cancer state and are not influenced by the presence of other health conditions.

Larger studies are needed to further investigate the correlation of NOHA normalization with attainment of pCR among those undergoing curative intent NT due to the significant gap in blood‐based strategies to direct treatment decision making and optimization in the TNBC NT setting. Specifically, previous work has examined the utility of circulating tumor DNA (ctDNA) and circulating tumor cells (CTCs) in the neoadjuvant management of TNBC. Although these studies have shown that the presence of ctDNA or CTC following NT is predictive of disease recurrence, and detection of CTC at the outset of NT is predictive of treatment outcome [21], there is limited evidence supporting the use of these technologies as measures of longitudinal treatment response and as means to optimize treatment decision making in the NT setting [22, 23, 24, 25]. Further, other extensively studied antigen‐based biomarkers, including CA 15–3, CEA and CA 125, are known to be of limited utility as longitudinal measures of NT response in the management of breast cancer [26].

The findings in this study corroborate our previous data showing that blood NOHA levels are very low (< 4 nM) in individuals with ER− disease compared with healthy individuals, who typically exhibit NOHA levels > 8nM [8, 10]. Our future directions include evaluation of NOHA as a complement to available breast cancer screening or surveillance modalities. Specifically, NOHA may serve as an accessible screening tool among women at increased risk for TNBC, that is, those with *BRCA1* mutations and African Americans, and as a measure of disease recurrence among breast cancer survivors. Finally, our emerging data on NOHA in breast cancer, supports future studies exploring the role of the NOHA biomarker in TNBC drug development [27].

In conclusion, this pilot study demonstrates that plasma NOHA concentrations increase progressively toward healthy‐control levels during and after NT in TNBC, independent of pCR. These findings support NOHA as a potential marker of disease presence and treatment exposure, but larger, adequately powered studies are required to determine whether serial NOHA monitoring can inform individualized treatment adaptation or improve outcomes in high‐risk populations.

## Author Contributions

**Srinidi Mohan:** conceptualization, investigation, funding acquisition, methodology, writing – review and editing, project administration, supervision, resources, data curation, writing – original draft, formal analysis. **Susan Miesfeldt:** conceptualization, investigation, funding acquisition, writing – review and editing, formal analysis, project administration, data curation, supervision, resources, methodology, writing – original draft. **Rachel J. Buchsbaum:** investigation, writing – review and editing, resources. **Cara L. Frankenfeld:** formal analysis, writing – review and editing, resources, supervision. **John DiPalazzo:** writing – review and editing, visualization, formal analysis, data curation. **Jamie G. Saunders:** writing – review and editing, project administration, data curation, supervision. **Shelby E. Monahan:** investigation. **Brittany Y. Bertaux:** writing – review and editing, resources, investigation. **Douglas W. Van Pelt:** writing – original draft, writing – review and editing, visualization, formal analysis.

## Funding

The research reported here was supported by the Maine Cancer Foundation, Portland, Maine USA, and by NIGMS of the National Institutes of Health under award number U54GM115516, “Northern New England Clinical and Translational Research Network”, Bethesda, Maryland USA.

## Ethics Statement

The MMC Institutional Review Board (IRB) approved the study protocol and procedures. All procedures performed in this study were in accordance with the ethical standards of the institutional research committees and with the 1964 Helsinki declaration and its later amendments or comparable ethical standards. Informed consent was obtained from all study participants.

## Conflicts of Interest

Srinidi Mohan and Susan Miesfeldt are co‐founders of SatyaDx which is developing the NOHA biomarker. The University of New England owns two patents for the biomarker (US Utility Patent numbers 10,073,099 and 11,009,507). The other authors declare no conflicts of interest.

## Supporting information


**Figure S1:** Longitudinal plasma NOHA concentration across all subjects (*n* = 31). Pre‐NT = within 7 days prior to NT initiation, sample *n* = 29; NT 1 = 4–8 weeks after start of NT, sample *n* = 28; NT 2 = 10–14 weeks after the start of NT, sample *n* = 27; Post‐NT = 1–3 weeks after completion of NT, sample *n* = 27; Post‐surgery = 2–7 weeks after surgery, sample *n* = 25.
**Table S1:** Effect on pre‐treatment NOHA Results of univariable regression models used to assess association between pre‐treatment NOHA levels and age, race, comorbidities, cancer grade, and stage. *p* < 0.05 was considered statistically significant.

## Data Availability

The data that support the findings of this study are available from the corresponding author upon reasonable request.
