# Supplementary material for: Nw‐Hydroxy L‐Arginine (NOHA): A Promising Biomarker of Response to Neoadjuvant Therapy in the Management of Triple Negative Breast Cancer
Source: Cancer Rep (Hoboken). 2026 Jul 8;9(7):e70609. doi: 10.1002/cnr2.70609 (PMC13345984; doi:10.1002/cnr2.70609)
Supplement: Supplementary file 1 — Figure S1: Longitudinal plasma NOHA concentration across all subjects (n = 31). Pre‐NT = within 7 days prior to NT initiation, sample n = 29; NT 1 = 4–8 weeks after start of NT, sample n = 28; NT 2 = 10–14 weeks after the start of NT, sample n = 27; Post‐NT = 1–3 weeks after completion of NT, sample n = 27; Post‐surgery = 2–7 weeks after surgery, sample n = 25. Table S1: Effect on pre‐treatment NOHA Results of univariable regression models used to assess association between pre‐treatment NOHA levels and age, race, comorbidities, cancer grade, and stage. p < 0.05 was considered statistically significant. [file CNR2-9-e70609-s001.docx]

**Supplementary material**


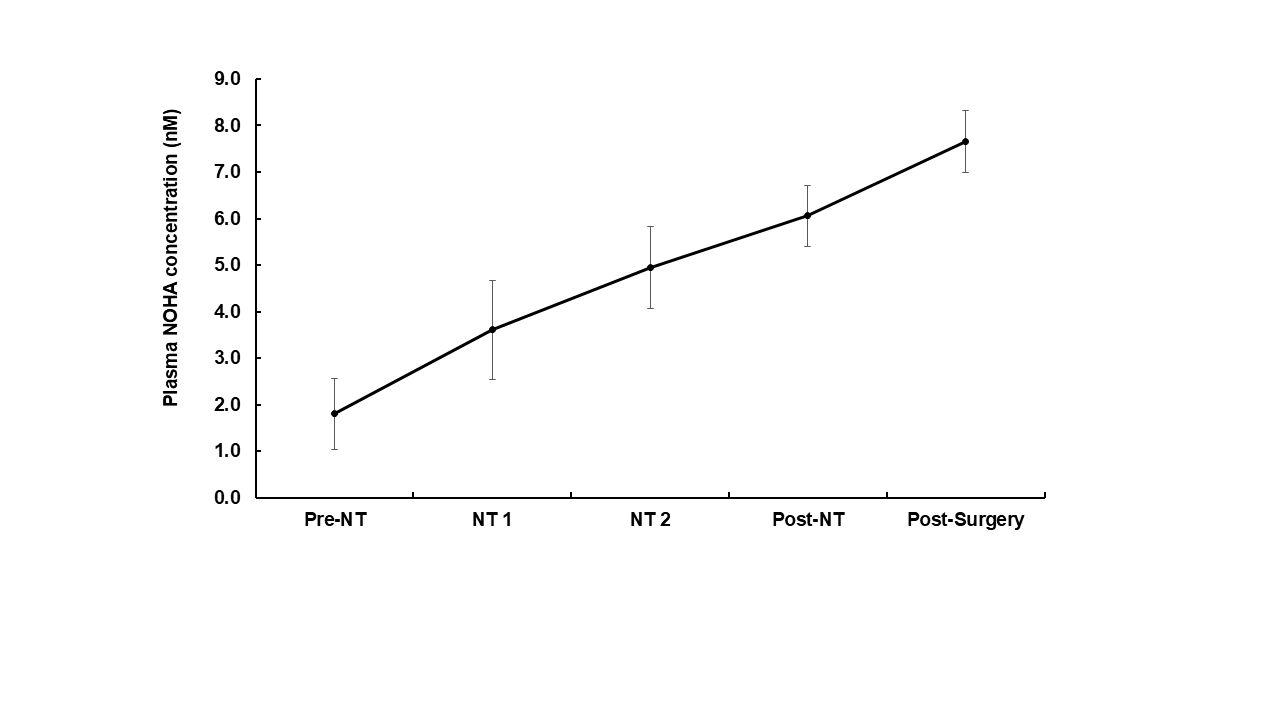


**Supplemental Figure 1: Longitudinal plasma NOHA concentration across all subjects (n=31).** Pre-NT = within 7 days prior to NT initiation, sample n=29; NT 1 = 4-8 weeks after start of NT, sample n=28; NT 2 = 10-14 weeks after the start of NT, sample n=27; Post-NT = 1-3 weeks after completion of NT, sample n=27; Post-surgery = 2-7 weeks after surgery, sample n=25.

| **Supplemental Table 1: Effect on pre-treatment NOHA** | | | | |
| --- | --- | --- | --- | --- |
| **Characteristic** | **n*** | **Beta** | **95% CI** | **P-value** |
| Age (years) |  |  |  |  |
| >50 | 17 | - | - |  |
| <50 | 12 | 0.43 | -0.15, 1.0 | 0.14 |
| Comorbidities |  |  |  |  |
| None | 15 | - | - |  |
| ≥1 | 14 | 0.20 | -0.39, 0.78 | 0.5 |
| Hypertension |  |  |  |  |
| No | 16 | - | - |  |
| Yes | 13 | 0.30 | -0.28, 0.88 | 0.3 |
| Stage |  |  |  |  |
| I or II | 19 | - | - |  |
| III | 10 | -0.27 | -0.88, 0.35 | 0.4 |
